# Mental distress among university students in Ethiopia: a cross sectional survey

**DOI:** 10.11604/pamj.2013.15.95.2173

**Published:** 2013-07-11

**Authors:** Yadeta Dessie, Jemal Ebrahim, Tadesse Awoke

**Affiliations:** 1 Deparement of Public Health, Colleges of Health and Medical Sciences, Haramaya University,Harar, Ethiopia; 2 Department of Psychiatry, Colleges of Health and Medical Sciences, Haramaya University, Harar, Ethiopia; 3 Department of Epidemiology and Biostatistics, Institute of Public Health, College of Medicine and Health Science, University of Gondar, Gondar, Ethiopia

**Keywords:** Mental distress, substance Use, university, tertiary education

## Abstract

**Introduction:**

Mental distress is becoming a common health problem among university students. There is limited information in this regard in Ethiopia. This study was aimed to determine the prevalence and associated factors of mental distress among students in Adama University.

**Methods:**

A cross-sectional study was conducted in March2011. Four hundred and thirteen students were participated in the study. Simple random sampling technique was applied to select the study participants. Self-Reporting Questionnaire-20(SRQ-20) was used to assess the mental distress. Respondents who had a score of eleven and above on the SRQ-20 were considered as mentally distressed.

**Results:**

The prevalence of mental distress was 21.6%. Family history of mental illness (AOR=2.30, 95%CI: 1.10 - 4.81), frequent conflicts with fellows (AOR=2.26, 95%CI: 1.10 - 4.85), Khat (Catha Edulis) chewing (AOR=2.23, 95% CI: 1.14 - 4.35) and not attending religious program regularly were factors associated with mental distress. Being in second year of their education less likely associated (AOR=0.41, 95%CI: 0.18 - 0.91) with mental distress.

**Conclusion:**

About one fifth of the students were found to be mentally distressed. Designing prevention sand treatments programs addressing the identified factors is important.

## Introduction

Mental distress is a mental health problem which manifests with different levels of depressive, anxiety, panic or somatic symptoms.It also presents with confused emotions, hallucination and related symptoms without actually being ill in a medical sense [1, 2]. This problem has a direct and indirect effects on the individual's psychology, social functioning and affects many aspect of life including relationships, work and health [3, 4].

Significant proportion of the world population is affected by mental distress of which tertiary education students are the once [5–8]. Studies revealed that more than half of students in different countries like Singapore and United States of America (USA) had experienced emotional distress [9, 10]. In the same aspect, 41.9% of students in Malaysia and 53.0% in Australian reported to have psychological distress [11, 12].

Various factors were reported to be associated with the development of mental distress among university students. Separation from pre-existing social support, frustration with academic challenges, social problems, and threats due to high expectations from parents; teachers were reported attributes of mental distress which could present variably in different contexts [13–18].

In Ethiopia, mental disorders was reported to account for 11% of the total burden of diseases [19]. Though limited and inconclusive, a mental distress prevalence of 32.6% to 49.1%was reported among university students in Ethiopia [20, 21]. Despite mental health problem was included in national health policy of Ethiopia, interventions against the problem are limited. The main reason is the lack of data on the extent of the problem [22]. This study was aimed to determine the prevalence of mental distress and identify the contributing factors among students in Adama University, Ethiopia.

## Methods

### Study Setting

This study was a cross-sectional survey conducted among undergraduate students of Adama University; Eastern Ethiopia. It was carried out from March 7-30; 2011. During the study period, the university had more than nine thousand regular undergraduate students of whom twenty percent were females. The study sample size was determined by a single population proportion formula with the assumptions of 95% level of confidence, 5% margin of error, prevalence of metal distress 49% which was taken from previous study conducted in the country [20] and an added15% non-response rate. With this calculation, the final sample size was 442. Simple random sampling technique was applied to select the study subjects using the list of students from the university office of registrar as a sampling frame.

### Data collection

A self-administered structured questionnaire was used to collect the data. The questionnaire was derived from different literatures that included the socio-demographic characteristics, history of substance use, social issues related questions and questions addressing mental distress called Self-Reporting Questionnaire-20(SRQ-20). The questionnaire was first prepared in English and then translated to Amharic for data collection. The level of mental distress was measured using SRQ-20 items (with a 30 days recall period), which had been used previously for screening of common mental problems [23, 24]. The tool (SRQ'20 items) reflects the multidimensional nature of mental disorders. It includes somatic factors, depressive/anxiety symptoms, and cognitive/decreased energy factor. The tool was validated and used in the country [25]. In this study, a cut of point of11 and above was taken to classify to mentally distress which was also used previously [26].

### Data analysis

The data were entered into Epi-Info version 3.5.3 and transferred to SPSS (V16.0) for analysis. For testing the statistical significance, odds ratio with 95% Confidence Level was calculated for each independent variable against the dependent variable using the bivariate logistic regression. Multivariable logistic regression analysis was performed for those variable shown p-value of less than or equal to 0.05 in the bivariate analysis to control for the confounders and identify the independent factors. A p-value less than or equal 0.05 was used to declare the presence statistical significance.

### Ethical considerations

Ethical clearance was obtained from the Institutional Research Ethics Review Committee (IRERC) of University of Gonder. A letter introducing the objective of the study, and maintaining the confidentiality was attached as the cover page of the questionnaire. Participants were consented for participation in the study. The right to refuse was clearly stated in the letter if the respondent is not volunteer to participate in the study.

## Results

### Socio-demographic and related characteristics

Four hundred and thirteen respondents were studied which resulted in a repose rate of 97.3%. Respondents’ age ranges from 18 to 26 years, with a mean of 20.9 ± 1.5(SD) and (80.9%) were within 20-25 years. About 88% were males and most were Orthodox in their religion. A majority were in their second year and beyond in their education (90.0%) (Table 1). One hundred and seventy two (40.0%) had ever chewed Khat (Catha Edulis) and (33.3%) had chewed12 months prior to the study. Life time alcohol consumption and cigarette smoking were (37.9%) and 49 (11.4%) respectively. Fourteen (3.3%) had ever used sedative drugs (Table 2).

**Table 1:** Socio-demographic variables of respondents, Adama University Eastern Ethiopia April 2011

Characteristics			
		Frequency	%
**Age(years)**			
	≤19	76	16.7
	20-24	344	80.0
	≥25	10	2.3
**Gender**			
	Female	52	12.1
	Male	378	87.9
**Enrolment Year**			
	1^st^ year	50	11.6
	2^nd^ year	281	65.3
	3^rd^ year and above	99	23
**Religion**			
	Muslim	137	31.9
	Orthodox	183	42.6
	Protestant	100	23.3
	Others	10	2.3
**Family Marital Condition**			
	Living together	389	90.5
	Not living together	41	9.5
**Have boy or girl friend**			
	Yes	171	39.8
	No	259	60.2
**Have Close Friends**			
	Yes	365	84.9
	No	65	
**Relationship With Friends**			
	Satisfactory	362	98.2
	Not satisfactory	4	
**Relationship With Family**			
	Satisfactory	402	94.1
	Not satisfactory	25	5.9
**Family History Of Mental Illness**			
	Present	84	19.5
	Absent	346	80.5

**Table 2:** Distributions of substance use among students, Adama University, Eastern Ethiopia, April, 2011

Type of substance	Last 12 months N (%)	Ever users’ N (%)
**Khat use**		
Users	143 (33.3)	172 (40.0)
Non users	287(66.7)	258 (60.0)
**Alcohol use**		
Users	124 (28.8)	163 (37.9)
Non users	306 (71.2)	267 (62.1)
**Tobacco use**		
Users	37 (8.6)	49 (11.4)
Non users	393 (91.4)	381 (88.6)
**Shisha Use**		
Users	25 (5.8)	31 (7.2)
Non users	405 (94.2)	399 (92.8)
**Sedatives**		
Users	11 (2.6)	14 (3.3)
Non users	419 (97.4)	416 (96.7)

### Mental distress prevalence

**Figure 1:**
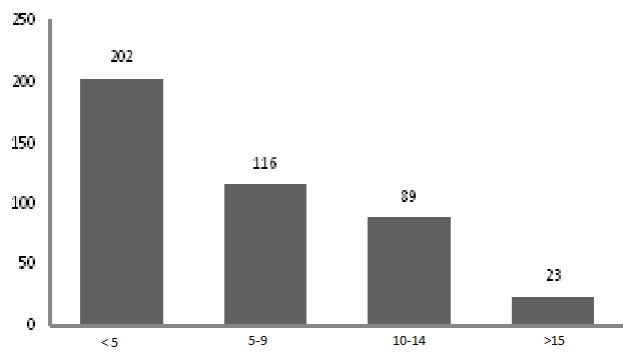
Distribution of SRQ-20 among students of Adama University, Eastern Ethiopia, 2011

Suicidal ideation one month before the study was 0.9%. The distribution of SRQ-20 showed a mean score of 5.87 ± 4.82 ranging between 0 and 19 (Figure 1). The prevalence of mental distress among the student was 21.6%.

### Factors associate with mental distress

Different factors associated with mental distress were identified. A higher level of mental distress was reported among students who have had conflicts with their fellow son different personal and social issues (OR 95% CI=2.26 (1.10 - 4.85)). Reported family history of mental illness was significantly associated with mental distress (OR with 95% CI=2.30 (1.10 - 4.81)) and those who had history of Khat chewing were more likely mentally distressed (OR=2.23; 95% CI=1.14-4.35). Being in second years of education was found to be a protective factors (AOR=0.41, 95% CI=0.18 - 0.91)and mental distress was low among those regular religious programs attenders, irrespective of what their religion (Table 3).

**Table 3:** Factors associated with mental distress among Adama University students, eastern Ethiopia, April 2011

Characteristics	Mental Distress		COR (95% CI)	AOR (95% CI)
**Year of enrolment**				
1^st^ year	11	39	0.65(0.30,1.44)	1.27(0.41,3.91)
2^nd^ year	52	229	0.52(0.31,0.88)	0.41(0.18,0.91)*
3^rd^ year and above	30	69	1.00	1.00
**Practice of religion**				
Always	6	19	1.00	1.00
Often	23	67	10.9(4.3,28.0)**	5.64(1.98,16.1)*
Some times	29	52	17.8(7.0,45.04)*	16.11(5.6,46.3)*
Rarely	35	27	41.3(15.9,107.3	32.7(11.2,95.7)*
**Family history of mental illness**				
Yes	27	57	2.0(1.18,3.42)	2.30(1.10,4.81)*
No	66	280	1.00	1.00
**Conflicts with fellows**				
Yes	33	51	3.08(1.84,5.18)	2.50(1.20,5.30)*
No	60	286	1.00	1.00
**Department choice**				
Preferred	39	209	1.00	1.00
Not preferred	54	128	2.26(1.42,3.61)	1.13(0.42,3.0)
**Interested to the department**				
Yes	40	207	1.00	
No	53	130	2.11(1.33,3.36)	1.79(0.65,4.88)
**Ever chewed Khat**				
Yes	59	126	2.91(1.81,4.7)	2.23(1.14,4.35)*
No	34	211	1.00	1.00
**Ever use alcohol**				
Yes	46	117	1.85(0.89,3.84)	0.73(0.35,1.51)
No	47	220	1.00	1.00
**Ever use tobacco**				
Yes	19	30	2.63(1.40,4.93)	0.97(0.42,2.24)
No	74	307	1.00	1.00
**Ever used Shisha**				
Yes	18	13	5.98(2.8,12.74)*	1.90(0.71,4.98)
No	75	324	1.00	1.00
**Ever used Sedatives**				
Yes	9	5	1.72(1.07,2.75)	1.38(0.32,5.93)
No	84	332	1.00	1.00

* Statistically significant at p<0.05

## Discussion

About one fifth (21.6%) of the study participants were mentally distressed. This finding is lower than what were reported from Malaysia, Spain, United Kingdom, Singapore, USA and Australia where a prevalence of 30%-57% of mental distresses were reported [9–12, 27–28]. The difference could be attributed to the socioeconomic, cultural and environmental factors. Further, most of these studies were done among medical schools students where the medical education environment is thought to be stressful and contributes to emotional and psychological disturbances [9, 21, 29]. Less, comparable and higher proportions of mental distresses when compared to the current study were reported among various group of populations including university students in Ethiopia [20–21–30–31].

The likelihood of mental distress was higher among ever Khat chewer. This built on what was reported by Damena and his colleagues where Khat chewing was found to be significant predictor of mental distress [5]. This issue is interwoven due to the fact that substance use is related to different facets of health problems. Moreover, because this study is a cross-sectional, it is difficult to conspicuously identify in which direction the causality is prevailing and it is beneficial to consider interventions addressing both. Though, it is not simple to give sound explanation, one important finding in this research is that, frequent conflicts due to different reasons was associated with higher level of mental distress. Earlier study also reported this phenomenon [20]. This might be resulted from the fact that university life where students live together in a group and a small to big conflicts might result in a stressful situation.

Religious teachings and advises help in stress management and as well facilitate the development of adaptive behaviors of individual's life [9, 20, 32–35]. In line with these, in our study, students’ involved in more frequent religious programs were less likely mentally distressed. Those in second year of education were less depressed and this can be seen in light of the fact students are usually s in their academic training as they come out of the frustrations of first year education. But, in third year and above again they commonly start to exercise what their senior peers are doing like using substance which could have an effect on mental distress.

In the study, parent marital condition, ethnicity, and gender of the respondents were not significantly associated with mental distress and similar findings were also reported [20, 21]. Though parental marital status was not significantly associated, investigating the time and process of parental separation or divorce which we were not addressed in this study needs further research.

This study was not without limitations. Reports for some of the questions were past history or encounters which are prone to recall bias. Variables like Khat chewing and other substances are by nature a sensitive issue and social desirability bias is unavoidable.

## Conclusion

About one fifth (21.6%) of the university students had mental distress. The likelihood of having mental distress were higher among students who had family history of mental illness, never attended religious programs, frequent conflicts with their fellows and chew khat. Designing preventions and treatments programs tailored to the contexts is essential.
